# Sex as a biological variable in determining the metabolic changes influencing acute ischemic stroke outcomes—Where is the data: A systematic review

**DOI:** 10.3389/fneur.2022.1026431

**Published:** 2022-11-24

**Authors:** Layne Dylla, Hannah M. Higgins, Christi Piper, Sharon N. Poisson, Paco S. Herson, Andrew A. Monte

**Affiliations:** ^1^Department of Emergency Medicine, University of Colorado School of Medicine, Aurora, CO, United States; ^2^Strauss Health Sciences Library, University of Colorado School of Medicine, Aurora, CO, United States; ^3^Department of Neurology, University of Colorado School of Medicine, Aurora, CO, United States; ^4^Department of Neurological Surgery, The Ohio State University, Columbus, OH, United States

**Keywords:** metabolomics, ischemic stroke, sex-differences, neurological outcome, stroke

## Abstract

Women continue to face a greater lifetime morbidity and mortality from stroke and have been shown to respond differently to stroke treatments compared to men. Since 2016, updated National Institutes of Health (NIH) policies require research studies to consider sex as a biological variable. However, the way in which this policy affects study design, analysis, and reporting is variable, with few studies performing and reporting a subgroup analysis based on biological sex. In acute ischemic stroke, the underlying biological explanation for sex-based differences in patient outcomes and response to treatments remains understudied. We performed a systematic review of preclinical and clinical research studies that explored sex differences in the metabolic response to acute ischemic stroke as it relates to neurological outcomes. Through a literature search in Ovid Medline, Embase, and Web of Science, 1,004 potential references were identified for screening. After abstract and full-text review, we identified only two studies which assessed metabolic response to acute ischemic stroke (within 72 h of last known well) and neurological outcome [Barthel Index, modified Rankin Scale (mRS) or an equivalent in preclinical models] and reported results based on biological sex. One article was a preclinical rat model and the other a clinical cohort study. In both studies, metabolites involved in amino acid metabolism, energy metabolism, fat metabolism, or oxidative stress were identified. We review these results and link to additional articles that use metabolomics to identify metabolites differentially expressed by sex or regulated based on stroke outcomes, but not both. The results of this systematic review should not only help identify targets in need of further investigation to improve the understanding of sex differences in the pathophysiology of acute ischemic stroke, but also highlight the critical need to expand the incorporation of sex as a biological variable in acute stroke research beyond simply including both sexes and reporting the proportion of males/females in each population studied.

## Introduction

Stroke remains a leading cause of death and disability worldwide with the majority being ischemic in nature (1). Despite many advances in stroke care, females experiencing acute ischemic stroke (AIS) are at greater lifetime risk of AIS and suffer greater morbidity and mortality from AIS compared to males (1–3). Additionally, females have been shown to respond differently to existing therapies and in clinical trials to novel therapeutic approaches (4, 5). The reasons for this are not well-understood. However, it is likely multifactorial, with contributions from stroke at increased age in females, differences in underlying risk factors, underlying differences in the cell death response to stroke, underlying differences in the immune response, and other unidentified differences in the metabolic response to stroke. Many of these variances have been previously reviewed apart from underlying differences in the metabolic response to ischemic stroke (2, 6–9).

The pathophysiology of stroke and the resulting total infarct volume are metabolically active events. After the onset of ischemia, there is a complex set of molecular events that begin within minutes to hours and ultimately result in neuronal cell death. These events are initially driven by decreased blood flow and hypoxia, but are quickly followed by tissue depolarization and a subsequent increase in metabolic rate. Coupled with an already low oxygen supply and compromised cerebral blood flow, this creates a hypoxic environment that perpetuates tissue destruction and infarct size (2, 3). Tissue damage resulting from this hypoxia is mediated in part by a shift to anaerobic metabolism with increased lactic acid production. This helps facilitate the production of excess amounts of oxygen and nitrogen free radicals. Ultimately, these processes lead to lipid peroxidation, protein denaturation, DNA damage and activation of apoptotic pathways that eventually result in cell death (4, 5).

Despite a basic understanding of the pathophysiology of stroke, the exact mechanisms and metabolic response to ischemic stroke have not been fully elucidated. Improved understanding of this metabolic response and identification of potential mechanisms to salvage penumbral tissue may lead to future therapeutic advances. Currently, treatment for acute ischemic stroke is limited to either intravenous thrombolysis with a tissue plasminogen activator (tPA) such as Alteplase or Tenecteplase, or endovascular therapy with direct clot lysis or mechanical thrombectomy. These acute therapies have limited treatment windows and it is estimated that only 10–15% of patients presenting to either a comprehensive or primary stroke center receive tPA with only 1–4% receiving endovascular therapy (6). Furthermore, despite advances in stroke care, women continue to be less likely to receive either tPA or endovascular therapy [reviewed in (10)].

Metabolomics – the study of small downstream targets that reflect upstream metabolic processes – has the power to inform the biology of acute ischemic stroke and lead to novel therapeutics. Metabolomics has been used to differentiate between types of stroke (hemorrhagic, ischemic and ischemic subtypes) (10–12), transient ischemic attack and ischemic stroke (13), and those with an ischemic stroke compared to controls (14–19). Metabolomic analyses have also been used to identify metabolic changes that predict infarct volume (20), functional outcome after stroke (21, 22), and post-stroke depression (23–25). Extensive reviews of general metabolomic analysis of stroke have been previously published. However, despite the growing use of metabolomics to inform understanding of the underlying biology driving acute ischemic stroke, few studies evaluate potential sex-based differences in the metabolic response to stroke that could drive the disparate outcomes faced by women. The objective of this review is to describe current evidence with regards to sex-based differences in the metabolic response to stroke in terms of functional outcomes [i.e., National Institutes of Health Stroke Scale (NIHSS) and mRS or equivalent functional testing in preclinical models].

## Methods

This systematic review was conducted with close attention to the PRISMA (Preferred Reporting Items for Systematic reviews and Meta-Analyses) guidelines (26) (see Supplementary Table 1 for complete PRISMA 2020 Checklist).

### Literature search

A comprehensive literature search was designed and performed by an experienced medical librarian (CP) in May 2022 for the concepts of metabolomics and ischemic stroke. Relevant publications were identified by searching the following databases with a combination of standardized index terms and keywords: Ovid MEDLINE ALL (1946 to May 20, 2022), Embase (*via* Elsevier, 1947 to present), and Web of Science Core Collection (*via* Thomson Reuters, including Science Citation Index Expanded 1974 to present, and Social Sciences Citation Index 1974 to present). The search was constructed for Ovid MEDLINE and translated to the additional databases. All results were exported to and deduplicated in EndNote 20. Covidence systematic review software (Veritas Health Innovation) was used for screening and full text review. See Supplementary Table 2 for search details.

### Inclusion criteria

Eligibility criteria was determined *a priori*. Included studies provided data on the sex differences in the metabolic response to acute ischemic stroke and functional outcome. Conference abstracts were excluded. Included studies met the definition of acute ischemic stroke with data collected within 72 h of last known well. Subgroup analysis by sex for differential expression of metabolites and/or a functional outcome (i.e., NIHSS, mRS, Barthel Index, or other functional outcome defined in preclinical models) had to be reported. For targeted metabolic studies, a metabolite was defined as a small molecular substance involved in metabolic processes as previously identified in the Human Metabolome Database (27, 28).

### Study selection

Citations and abstracts were uploaded in Covidence for study selection. *Two* authors (LD and HMH) independently screened all titles and abstracts and subsequent full-text articles. Data were compiled and consensus was reached by discussion on any disagreements for exclusion. Quality assessment of studies was done using the Collaborative Approach to Meta-Analysis and Review of Animal Data from Experimental Studies (CAMARADES) protocol for animal data (28) and the Newcastle-Ottawa Scale (NOS) for clinical data (29). Quality assessment was performed by two authors (LD and HMH). Data extraction was completed by a single author (LD).

## Results

### Study characteristics

**Figure 1:**
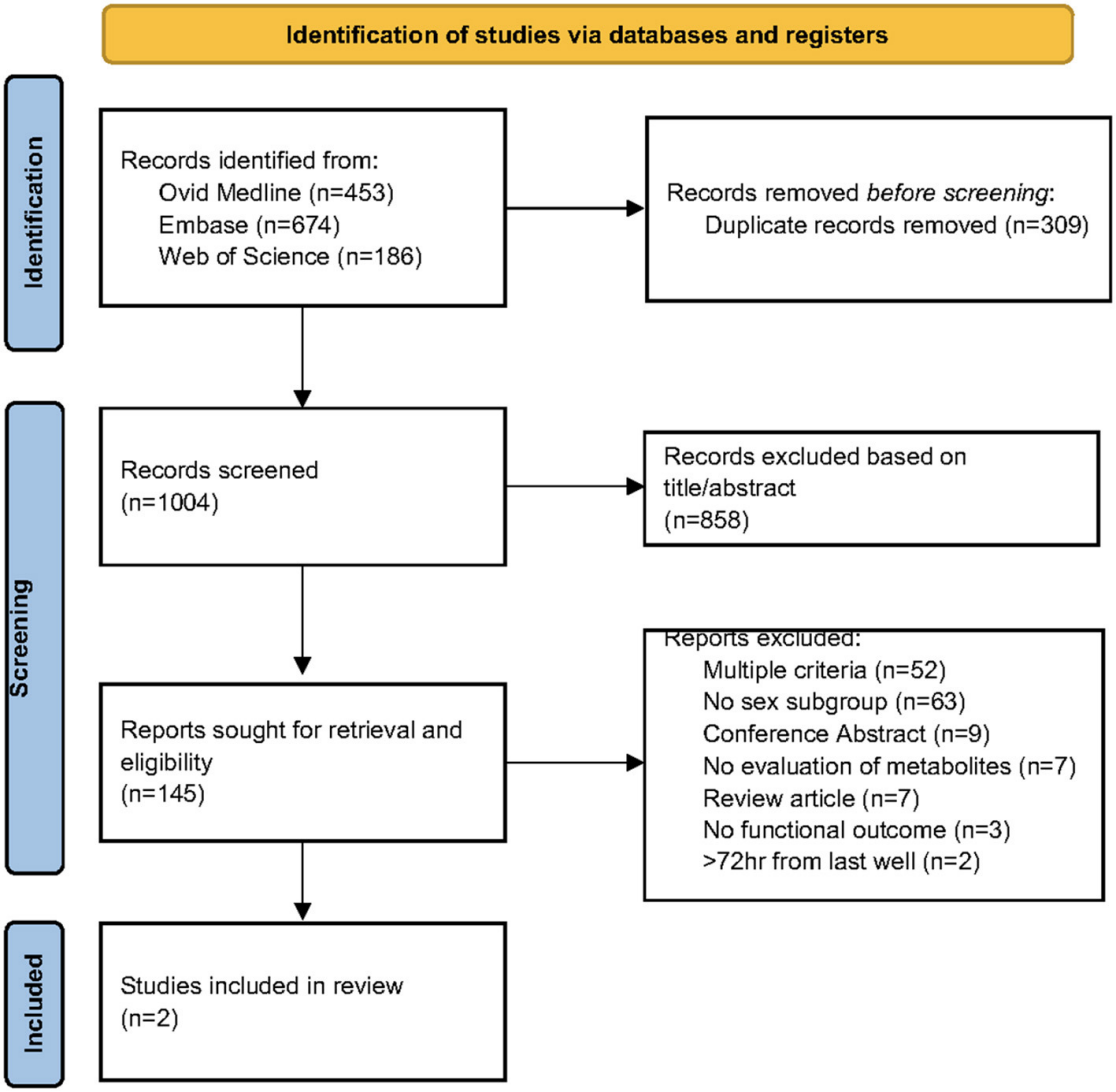
PRISMA diagram.

One thousand four study titles and abstracts were screened for inclusion. One hundred forty-six full-text studies were assessed for eligibility with the majority being excluded for more than one reason (usually lack of sex-subgroup reporting of outcomes and analysis of stroke patients more than 72 h from last known well) (see Figure 1 for PRISMA diagram). Of the two included studies (Table 1), one was a preclinical middle cerebral artery occlusion (MCAO) rodent model of acute ischemic stroke (22) and the other a clinical study of 45 acute first-ever ischemic stroke patients and 40 healthy controls who were part of an existing longitudinal study in Norway (30). The preclinical study used ^1^H NMR (nuclear magnetic resonance) spectroscopy of serum samples to determine the metabolic response to MCAO and a 10-day pretreatment with Huang-Lian-Jie-Du Decoctin, a traditional Chinese medicine used to treat cerebral ischemic/reperfusion injury, compared to controls. In the clinical study, serum samples were collected within < 72 h (most were collected within 24 h) from stroke onset. Targeted analysis of tryptophan (an essential amino acid metabolized to several neuroactive metabolites *via* the kynurenine pathway) was performed using high performance liquid chromatography (HPLC) to identify metabolites associated with stroke and neurological outcomes.

**Table 1:** Summary of included studies.

**Study**	**Number subjects**	**Comparison groups**	**Metabolites with at least 20% decrease**	**Metabolites with at least 20% increase**	**Reference**
1	≥20 rats per group: a) Female Sham-operated b) Male Sham-operated	Female MCAO compared to female sham-operated	• β-hydroxybutyrate • Acetoacetate	• Valine • Alanine	Zhang et al. (22)
	c) Female MCAO d) Male MCAO e) Female HLJDD** f) Male HLJDD** g) Female negative control/placebo h) Male negative control/placebo	Male MCAO vs. male sham-operated	• Alanine • Acetoacetate • Glutamine • Pyruvate • Glucose • Betaine	• LDL/VLDL • Lactate • N-acetyl glycoproteins • O-acetyl glycoproteins • PUFA	
2	a) 45 first-ever acute ischemic stroke patients (27 male, 18 female; ages 37–85) b) 40 health controls (20 male, 20 females; ages 50–70)	Female stroke vs. male stroke	• Trp Index*	• Quinolinic Acid • Ratio quinolinic acid to kynurenic acid	Ormstad et al. (30)

^*^Trp index defined as relative concentrations: tryptophan/(tryptophan+valine+phenylalanine+isoleucine+leucine) x 100.

**HLJDD (Huang-Lian-Jie-Du Decoctin).

### Quality assessment

The preclinical analysis of sex-specific response to middle cerebral artery occlusion (MCAO) with or without treatment with Huang-Lian-Jie-Du Decoctin was determined to be of moderate-high quality with low risk of bias based on the CAMARADES approach which is specific to ischemia models and includes assessment of sample size calculations, randomization and blinding, the use of anesthetic without intrinsic neuroprotective activity, and control of temperature during ischemic experiments (28). Zhang et al. (22) did not specify a sample size calculation and did not specifically state control of the temperature during the rodent experiments and only received 8/10 points. The NOS scale was used to assess the quality and potential bias of the case control study assessing the impact of metabolic changes in acute ischemic stroke on functional outcomes. Ormstad et al. (30) was determined to be of overall moderate-high quality with moderate risk for bias (received 7/9 stars with no stars for comparability between cases and controls). Notably, authors attempted to age and sex match stroke cases with healthy controls. However, controls were significantly younger, and authors did not report on comorbidities within controls.

### Preclinical sex-differences in the metabolic response to MCAO

Few preclinical studies of ischemic stroke included both male and female rodents. In Zhang et al. (22), authors included adult male and female Sprague-Dawley rats who were pretreated with 10 days of Huang-Lian-Jie-Du Decoctin or placebo prior to middle cerebral artery occlusion (MCAO) or sham procedure (at least 20 animals per group with a total sample size of over 80 rats). In addition to metabolic analysis, rats were subjected to neurobehavioral testing by blinded observers using Longa's five-point scale to grade the neurological deficit from 0 (normal, no neurobehavioral dysfunction) to 4 (no autonomous activity and unconsciousness). The study found that both female and male rats treated with Huang-Lian-Jie-Du Decoctin prior to MCAO had less severe neurobehavioral dysfunction on the Longa's score (females with Huang-Lian-Jie-Du Decoctin - 0.67+/= 0.62 vs. 1.4 +/- 0.64; males with Huang-Lian-Jie-Du Decoctin 1.2 +/- 0.69 vs 2.1 +/-0.49). Twenty metabolites were identified to be explicitly different in males and females with MCAO. These compounds included: low-density lipoprotein (LDL)/very low-density lipoprotein (VLDL), PUFA, β-hydroxybutyrate, acetate, acetoacetate, glucose, pyruvate, citrate, malate, lactate, creatine, phosphocreatine, creatinine, choline, O-phosphocholine, phenylalanine, tyrosine, tryptophan, and N-acetylaspartic acid. These metabolites are commonly involved in oxidative stress, energy metabolism, fat metabolism, and amino acid metabolism likely playing key roles in the pathophysiology of acute ischemic stroke. Specifically, choline and O-phosphocholine are involved in oxidative stress and were both upregulated in male and female MCAO rats but showed greater reduction after pretreatment with Huang-Lian-Jie-Du Decoctin in females than males. Thus, Huang-Lian-Jie-Du Decoctin may be more protective against reactive oxygen species in female MCAO compared to male MCAO rats.

In terms of energy metabolism, lactate was increased in male, but not female MCAO rats, compared to control rats. Citrate, pyruvate, glucose, and malate were overall lower in MCAO rats compared to controls. These metabolites, in conjunction with lactate, are integral in energy metabolism and changes in concentration can reflect a shift from aerobic metabolism *via* the TCA cycle to anaerobic metabolism. In male MCAO rats, the almost 20% decrease in phosphocreatine and slight increase in creatine also supports a TCA cycle failure. Increased conversion of phosphocreatine to creatine can supply the high energy bond in phosphocreatine to create adenosine triphosphate (ATP). These data suggest that the imbalance of energy metabolism and the production of ATP may be more impacted in male MCAO rats compared to females MCAO rats, which did not demonstrate these same changes compared to female controls. Additionally, only male MCAO rats showed a >20% increase in metabolites involved in lipid fatty acid β-oxidation compared to controls (increased levels of LDL/VLDL, PUFA with a concomitant decrease in β-hydroxybutyrate, acetoacetate).

In contrast to the male-specific changes in energy and lipid metabolism, female MCAO rats exhibited a greater change in amino acid metabolism compared to controls, which was not seen in males. Specifically, female MCAO rats had higher levels of aromatic amino acids (phenylalanine, tyrosine, tryptophan) compared to female controls, while male MCAO rats exhibited no significant change compared to male controls. In females, MCAO rats also had slightly higher levels of N-acetylaspartic acid compared to controls whereas in males, MCAO rats showed a significant decrease compared to controls.

Despite multiple notable differences in metabolic response to MCAO between males and females, this study did not directly link these metabolic changes to different neurological outcomes. Rather, authors reported differential metabolomic profiles and differential neurological function independently. However, many of the differences in metabolic profiles support the relatively preserved neurological function after MCAO in female rats compared to males MCAO rats. The caveat in terms of translation to humans being that this study did not use aged rats. This allows for preserved estrogen effects in female rats that might not recapitulate the precise sex-differences in metabolic response to stroke in human post-menopausal women. While this study was an overall robust study in its design with the use of sham and placebo groups, comparison of serum and cerebral tissue metabolomic profiles, it remains limited by a lack of a priori sample size calculations and the need for further in-depth confirmation of the importance of the metabolic changes identified.

### Clinical sex-differences in metabolic response to acute ischemic stroke

**Figure 2:**
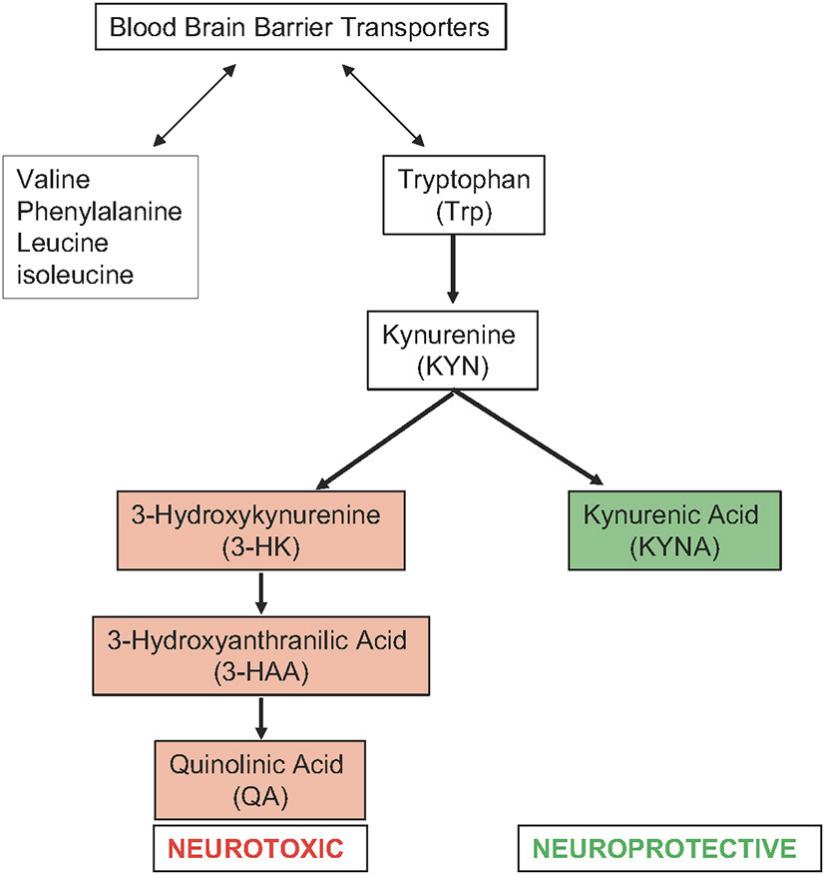
Tryptophan metabolism via the kynurenine pathway in acute ischemic stroke **(right)** and amino acids that compete with tryptophan for transport across the blood brain barrier **(left)**.

Like many preclinical models, which often do not report results based on biological sex when they do include both sexes, few clinical studies in stroke go beyond reporting cohort demographics (11). Ormstad et al. (30) assessed the role of amino acids (tryptophan, tyrosine) and their downstream metabolic targets in acute ischemic stroke (Figure 2: Tryptophan Metabolism). This longitudinal study compared the serum metabolome of 45 incident stroke patients within 72 h of onset of symptoms to 40 healthy controls. Neurological function was assessed using the Barthel Index 20 scale (graded from 0 to 20 with a higher score representing greater functional independence) at admission, which has been demonstrated to predict long term functional outcomes (31). The study found a negative correlation between the Barthel Index 20 Scale score and the ratio of kynurenine to tryptophan. The tryptophan level alone was positively correlated with the Barthel Index 20 scale score. This suggests that tryptophan metabolism *via* the kynurenine pathway is associated with worse neurological function. Females with stroke also showed higher levels of the downstream kynurenine pathway metabolite quinolinic acid, higher levels of the ratio of quinolinic acid to kynurenic acid, and lower levels of a tryptophan index. The tryptophan index {100x[tryptophan/(tryptophan+valine+phenylalanine+leucine+isoleucine)]} reflects the balance between tryptophan and other amino acids that compete with tryptophan for transport across the blood brain barrier. Together, this would suggest that females have higher rates of metabolism *via* the kynurenine pathway which may correlate with worse neurological function. However, again, there was no sex-based subgroup analysis of functional outcome, nor was there a direct analysis of functional outcome as it related to tryptophan metabolism in male stroke patients compared to female stroke patients.

Despite the limitations of this study in terms of inference between neurological outcome and sex-specific changes in metabolic response to stroke, the study again highlights the importance of amino acid metabolism in stroke outcomes. Tryptophan is an essential amino acid that is metabolized *via* the kynurenine pathway into multiple metabolically active compounds with both neurotoxic and neuroprotective roles. Tryptophan is primarily converted to kynurenine. Kynurenine is subsequently metabolized *via* either a neuroprotective pathway, which dominates under normal conditions, or a neurotoxic pathway, which dominates in response to pathological conditions. Under pathological conditions, high levels of proinflammatory cytokines stimulate the conversion of tryptophan to kynurenine. This shifts the pathway toward the neurotoxic branch (32). The neurotoxic branch is upregulated in pathological conditions, and produces 3-hydroxyantranilic acid, 3-hydroxykynurenine, and quinolinic acid among other metabolites. These neuroactive metabolites also play important roles in immune system regulation, neuroinflammation, oxidative stress, endothelial dysfunction, and cell death (32, 33). Thus, it is not surprising that the differences in tryptophan metabolism would correlate with neurological outcomes in stroke.

## Discussion

In this systematic review, we sought to identify studies that link metabolomics to disparate neurological outcomes in males and females with acute ischemic stroke. Despite a wealth of literature on metabolic differences between patients with acute ischemic stroke and controls or metabolic differences between patients with a good vs. poor outcome, few studies assess for differences in metabolic response to acute stroke that could drive the differences in outcomes faced by males and females. Only two studies, one clinical and one preclinical, performed a sex subgroup analysis of metabolic differences in response to stroke and differences in neurological outcome (22, 30). These studies identified amino acid metabolism as a potential metabolic process that differs between males and females and may be related to neurological outcome.

With limited treatment options currently available, and time limited use of those treatments, new neuroprotective strategies for stroke are needed. Prior attempts at neuroprotection have yielded few positive results and may fail in part due to lack of accounting for potential sex differences (34, 35). Additionally, while there have been significant declines in the morbidity and mortality from acute stroke in men, these advances have been less evident in women (6). After identifying over 1,000 articles including broad search terms such as “cerebrovascular disease,” “stroke,” “metabolite,” “metabolomics,” “degradomics,” “pharmacometabolomics,” and “biomarkers,” most preclinical studies were excluded due to including only male rodents. The use of male rodent models provides a well-controlled model but provides limited insight into the pathophysiology of acute ischemic stroke and viability of novel neuroprotective agents. In humans, ischemic stroke is most prevalent in elderly populations with one or more vascular comorbidities such as hyperglycemia, hypertension, dyslipidemia, and obesity–in fact, over 91% of stroke risk can be attributed to modifiable risk factors (6). Women typically live longer resulting in higher lifetime risk for stroke and are more likely to have an unfavorable outcome from stroke even after adjusting for age, potential risk factors, NIHSS at admission, prestroke mRS, tPA use, onset-to-door times, and stroke subtype (35–37). Thus, including aged mice of both sexes must undergird stroke models that inform the understanding of stroke in humans.

Our systematic review identified a single preclinical study that evaluated the metabolic response in male and female rats to a traditional Chinese medicine, Huang-Lian-Jie-Du Decoctin, and found that this compound improved neurological function in both male and female mice, with females having overall improved neurological function relative to males in both the placebo and Huang-Lian-Jie-Du Decoctin-treated rats (22). The group was able to identify numerous metabolites that were differentially expressed in male and female rats with MCAO that are commonly involved in oxidative stress, energy metabolism, fat metabolism, and amino acid metabolism. Potentially the most important finding was the identification the both choline and O-phosphocholine upregulation in male and female MCAO rats with greater reduction following Huang-Lian-Jie-Du Decoctin pretreatment in females compared to males. These metabolites play a key role in oxidative stress and the differential regulation by sex suggests that this Chinese medicine may be more protective against reactive oxygen species in females. While only a single targeted metabolic analysis in humans performed a sex-subgroup analysis of metabolic response and clinical outcomes, this group also identified key differences in amino acid metabolism, specifically tryptophan metabolism, based on biological sex that correlated with patient outcomes (30). Female stroke patients had upregulated neurotoxic kynurenine pathway metabolites, and upregulation of the kynurenine pathway was associated with worse neurological function in both males and females.

While our systematic review revealed a paucity of literature relating to sex differences in the metabolic response to acute ischemic stroke as it relates to patient outcomes, additional articles were identified that either assessed sex differences in the metabolic response to acute ischemic stroke or metabolic changes associated with functional outcomes. One article specifically addressed sex-based differences in the metabolic response to stroke (38). Analyzing the serum metabolic profiles of 18 male and 18 female acute ischemic stroke patients compared to matched controls revealed similar insights into the pathophysiology as presented in this systematic review. Fifty-five metabolites were differentially regulated in female stroke patients compared to controls, including amino acids involved in alanine and aspartate metabolism, tryptophan metabolism, leucine and isoleucine metabolism, and fatty acid metabolism. These metabolites also included numerous lipids that were involved in glycerophospholipid metabolism, arachidonic acid metabolism, and biosynthesis of unsaturated fatty acids. In males, only 39 metabolites were significantly altered. These included metabolites involved in valine, leucine, and isoleucine metabolism, pantothenate and CoA biosynthesis, and steroid hormone biosynthesis. Unfortunately, without ties to clinical outcomes it is unclear which, if any, of these metabolic processes may relate specifically to the different prognosis faced by women with stroke. However, with this limited data, it appears that amino acid metabolism, fatty acid metabolism, and steroid hormone biosynthesis may play roles in the sex differences in acute ischemic stroke. Further in-depth analyses are needed to confirm these findings and better elucidate the exact impact these metabolic processes have on clinical outcomes.

Numerous studies have deployed metabolomic assays to differentiate patients with acute ischemic stroke from controls [reviewed in: Au (39), Chumachenko et al. (40), Ke et al. (41), Qureshi et al. (42), and Zhang et al. (43)]. In terms of insight into functional outcomes and potential neuroprotective strategies, this approach has many advantages over traditional proteomics and genomics. Metabolomics is an extension of traditional ‘omics approaches that also analyzes changes in peptides, carbohydrates, lipids and amino acids. Metabolomics incorporates genetic changes that may predispose one to disease, developmental and pathological changes in the disease state, and exogenous responses to environmental and lifestyle factors (44) using a single scientific approach. Thus, metabolomics provides valuable insight that can lead to neuroprotective strategies for improved patient outcomes accounting for both endogenous and exogenous factors.

Despite the advantages of using metabolomics to identify differentially regulated processes related to stroke outcomes, few studies focus on this metric. In a large Italian study with over 200 available serum samples obtained within 24 h of treatment with tPA, Licari et al. (45) identified only three metabolites as significantly changed between subjects with a poor 3-month functional outcome (mRS ≥3) compared to those with a good 3-month functional outcome (mRS ≤ 2) (45). These included upregulation of 3-hydroxybutyrate, Subfraction ApoB LDL-3, and LDL3_PN in patients with a good functional outcome compared to those with a poor functional outcome. When the metabolomic profiling was interpreted in the context of metabolic networks and metabolite-metabolite connectivity, glutamine, tyrosine, leucine, lactate, acetone, acetate, and glycine were found to be significantly altered when comparing good and poor functional outcomes. In a separate study by Lee et al. (46) that assessed the association between plasma ceramides and neurological function after stroke, higher levels of ceramides at 24–72 h after acute ischemic stroke were also associated with poor neurological outcomes (mRS ≥2) (46). Plasma ceramides play an import role in cell membrane stability and cell signaling pathways involved in proliferation, differentiation, and death. However, again, there was no sex-specific subgroup analysis in either article.

Data presented in our systematic review identifies a key gap in the existing understanding of the metabolic response to acute ischemic stroke in terms of sex differences in functional outcomes. Few studies report outcomes and/or changes in metabolic profiles after stroke based on sex, even as a secondary subgroup analysis. Currently, to gain insight into this area, we are forced to consider limited data from studies that only assess sex differences or assess functional outcomes. Further targeted investigation of this area is needed to inform future neuroprotective strategies and improve outcomes for both men and women while accounting for sex as a biological variable. With this enhanced understanding of the underlying sex differences in metabolic response to stroke, novel neurotherapeutic approaches can be identified and modeled in preclinical studies of age mice of both sexes with hopes to ultimately translate these findings to humans.

## Limitations

This systematic review is limited by the identification of only two articles that met full inclusion criteria. However, this points to a key gap in the understanding of the biology of acute ischemic stroke. Few clinical studies perform a subgroup analysis by sex and/or focus on differences in metabolic response specifically related to neurological outcomes. Additionally, preclinical models focus on male rodents and fail to include sex-based analyses. In both cases, the studies were relatively small in sample size and quality assessment revealed key areas for improvement. However, the identified studies provide a key foundation for future work in sex-based differences in metabolic response to acute ischemic stroke that can provide insight into neurological outcomes.

## Conclusion

In conclusion, our systematic review identified amino acid metabolism as a potential mediator of different neurological outcomes in males and females with acute ischemic stroke. However, with only two articles out of over a 1,000 screened reporting sex-specific subgroup analyses and a neurological outcome, this review also identifies a key area ripe for future research.

## Data availability statement

The original contributions presented in the study are included in the article/Supplementary material, further inquiries can be directed to the corresponding author.

## Author contributions

LD was responsible for the initial conceptualization of the review. CP was responsible for literature search and article compilation. LD and HH were responsible for article screening, selection, and data collection. All authors contributed to the article and approved the submitted version.

## Funding

This work was supported in part by a National Institutes of Health Building Interdisciplinary Research Careers in Women's Health K12-HD057022 (PI: Judith Regensteiner) and an American Heart Association Career Development Award (AHA CDA #19CDA34660039; PI: LD).

## Conflict of interest

The authors declare that the research was conducted in the absence of any commercial or financial relationships that could be construed as a potential conflict of interest.

## Publisher's note

All claims expressed in this article are solely those of the authors and do not necessarily represent those of their affiliated organizations, or those of the publisher, the editors and the reviewers. Any product that may be evaluated in this article, or claim that may be made by its manufacturer, is not guaranteed or endorsed by the publisher.
