# Supplementary material for: Sex as a biological variable in determining the metabolic changes influencing acute ischemic stroke outcomes—Where is the data: A systematic review
Source: Front Neurol. 2022 Nov 24;13:1026431. doi: 10.3389/fneur.2022.1026431 (PMC9729945; doi:10.3389/fneur.2022.1026431)
Supplement: Supplementary file 2 [file Data_Sheet_2.pdf]

Search Strategies

Search Summary:

Database	No. Results
Ovid Medline	453
Embase	674
Web of Science	186
Total	1313
Duplicates removed	307
Unique articles to review	1006

MEDLINE (via Ovid MEDLINE® ALL, 1946 to present)		
Search date: May 20, 2022		
Search No.	Search Strategy	Results
1	exp "Ischemic Stroke"/	6575
2	(isch?emic adj3 stroke*).tw,kf.	74559
3	1 OR 2	75604
4	exp "Metabolomics"/ OR "Biomarkers"/me OR exp "Metabolome"/	91855
5	(Metabolomic* or Metabonomic* or degradomic* or nutrimetabolomic* or pharmacometabolomic* or pharmacometabonomic* or metabolome* OR ((metabolic OR metabolite*) ADJ3 biomarker*)).tw,kf.	49030
6	4 OR 5	116816
7	3 AND 6	453
8	remove duplicates from 7	453

Embase (via Elsevier, Embase.com, 1947 to present)		
Search date: May 20, 2022		
Search No.	Search Strategy	Results
#1	biological marker'/exp AND 'metabolism'/exp	137323
#2	metabolomics'/exp OR 'metabolome'/exp	53925
#3	(metabolomic* OR metabonomic* OR degradomic* OR nutrimetabolomic* OR pharmacometabolomic* OR pharmacometabonomic* OR metabolome* OR ((metabolic OR metabolite*) NEAR/3 biomarker*)):ti,ab,de	69743

#4	#1 OR #2 OR #3	198976
#5	ischemic stroke'/exp	13155
#6	(isch$emic NEAR/3 stroke*):ti,ab,de	125138
#7	#5 OR #6	125296
#8	#4 AND #7	1042
#9	#8 NOT [conference abstract]/lim	674

Web of Science Core Collection (via Clarivate Analytics, including Science Citation Index Expanded and Social Sciences Citation Index, 1974 to present)		
Search date: May 20, 2022		
#1	TS=(isch$emic NEAR/3 stroke*)	106891
#2	TS=(Metabolomic* or Metabonomic* or degradomic* or nutrimetabolomic* or pharmacometabolomic* or pharmacometabonomic* or metabolome* OR ((metabolic OR metabolite*) NEAR/3 biomarker*))	61959
#3	#1 AND #2	186
